# The Effects of Different UVA Photoperiods on the Growth Performance, Immune Responses, Antioxidant Status and Apoptosis-Related Gene Expression of the Pacific White Shrimp (*Penaeus vannamei*)

**DOI:** 10.3390/antibiotics11010037

**Published:** 2021-12-29

**Authors:** Xinyi Wang, Baoliang Liu, Xiaoqiang Gao, Xi Wang, Hongxu Li, Liang Xu, Guiming Wang, Kuifeng Zhao, Bin Huang

**Affiliations:** 1 Key Laboratory for Sustainable Development of Marine Fisheries, Ministry of Agriculture, Qingdao Key Laboratory for Marine Fish Breeding and Biotechnology, Yellow Sea Fisheries Research Institute, Chinese Academy of Fishery Sciences, Qingdao 266071, China; wangxinyi961@163.com (X.W.); gaoxq@ysfri.ac.cn (X.G.); xixi9611@163.com (X.W.); 719104437@163.com (H.L.); xl18068582805@163.com (L.X.); 6534586@163.com (K.Z.); 575465w33@163.com (B.H.); 2 College of Fisheries and Life Science, Shanghai Ocean University, Shanghai 201306, China; 3 Yuhai Hongqi Ocean Engineering Co. LTD, Rizhao 276800, China; 4325642@163.com

**Keywords:** ultraviolet A (UVA), growth, immune parameters, antioxidant capacity, apoptosis-related gene, *Penaeus vannamei*

## Abstract

UVA is the most common type of solar UV radiation in aquatic environments; however, the effects it causes in shrimp farming in recirculating water systems (RAS) is unclear. Thus, the growth performance, immune responses, antioxidant status and apoptosis-related gene expression in Pacific white shrimp, *Penaeus vannamei* (body weight 9.56 ± 0.10 g), reared with 12L: 12D full spectrum light as background light under five UVA (peak at 400 nm) photoperiods (0L: 24D, 2L: 22D, 4L: 20D, 8L: 16D and 12L: 12D) at a light intensity of 1 W/m^2^ were investigated. The results showed that the 2L: 22D and 4L: 20D UVA photoperiods enhanced the growth performance and reduced the feed conversion ratio and the shrimp mortality. Shrimp exposed to UVA (2L: 22D and 4L: 20D) also displayed higher levels of hepatopancreas catalase (CAT), superoxide dismutase (SOD), acid phosphatase (ACP), phenol oxidase (PO) and lysozyme (LZM) compared to the 8L: 16D and 12L: 12D groups. The malondialdehyde (MDA) levels increased in line with the extension of the UVA irradiation time. The mRNA expression of apoptosis-related genes in all the UVA treatments were significantly higher than with the control treatment, except for the 2L: 22D group. The results of the 2L: 22D and 4L: 20D treatments were significantly higher than those of the control group, except for LGBP. In conclusion, 2L: 22D and 4L: 20D UVA photoperiods increased growth performance and decreased FCR, improved the innate immunity and antioxidant response and reduced the mortality rate in adult shrimp.

## 1. Introduction

Ultraviolet light is a crucial element on the natural spectrum and serves a variety of ecological purposes. Of the three spectral UVR bands (ultraviolet A, UVA, 320–400 nm; ultraviolet B, UVB, 280–320 nm, and ultraviolet C, UVC, 200–280 nm), only UVA and UVB can enter the water column. Although UVC is extremely detrimental to organisms, the most harmful wavelengths are usually absorbed by the stratospheric ozone and, hence, never reach the Earth’s surface [1,2]. In recent decades, due to climate challenges such as global warming and ozone layer depletion, the amount of ambient UV radiation reaching the Earth’s surface has grown, which has ramifications for ecosystems. The impact of organisms’ exposure to ambient UV light is frequently studied, such as the evaluation of the physiological and behavioral responses of *Girella laevifrons* [3]. Browman et al. [4] reported that UVA and polarized light purportedly increase the visibility of prey by enhancing target contrast for fishes that can perceive them. Alves et al. [5] described the harmful effects of UVA and UVB radiations in fish at different lifecycle stages, including embryo, larvae, juveniles and adults. Groff et al. [6] reported that UV radiation caused DNA damage in *Colossoma macropomum* and *Arapaima gigas*, as detected by Comet assay. However, there have been few studies on prawns. Bok et al. [7] reported that *Haptosquilla trispinosa* featured polychromatic ultraviolet sensitivity. Salo et al. [8] found that UVA is required for a range of functions in animal creatures, including as a powerful modulator of fish immune defense. Rakete et al. [9] reported that UVA led to protein aggregation through ascorbate oxidation in humans. Additionally, according to previous research in our laboratory, the full spectrum plus a UVA light environment was most conducive to the development of *P. vannamei* [10].

*Penaeus vannamei* is a Penaeidae species native to the Pacific Ocean’s eastern seaboard that has been cultured in intensive and semi-intensive systems all over the world [11]. *P. vannamei* is the most important cultured shrimp species in China, which is a global leader in shrimp production. Indoor recirculation aquaculture systems have advanced quickly in recent years and have shown promise for long-term aquaculture system development in China. Nevertheless, aquaculture light environments have become a rising challenge as a result of the rise of industrialization and intensive aquaculture. Indoor factory culture, in contrast to traditional wide pond farming, restricts natural light. Furthermore, previous research has revealed that shrimps are extremely light-sensitive and that light directly or indirectly affects their development, feeding, growth and survival [12,13]. At the present time, supplemental UVA is rare in prawn factory farming.

The aim of this study was to investigate how varied UVA photoperiod supplements affected the growth performance, immune responses, antioxidant status and apoptosis-related gene expression of shrimp. This study’s findings will be used to improve artificial light supplementing approaches in shrimp farming.

## 2. Results

### 2.1. Growth Performance

The results on the effects of different UVA photoperiods on the growth performance of shrimp are presented in Table 1. The results showed that the growth rate and SGR of 2L:22D and 4L:20D groups were significantly higher than in the control group (*p* < 0.05), whereas there were no significant differences between the above two groups. Furthermore, the weight growth rate and SGR were significantly lowered (*p* < 0.05) in 8L:16D and 12L:12D groups compared to the control group. The mortality and FCR of the 2L:22D and 4L:20D groups were significantly lower than those of the other groups (*p* < 0.05); nevertheless, no significant differences were observed in terms of mortality and FCR in the above two groups. The mortality and FCR of the 8L:16D and 12L:12D groups were significantly higher than the control (*p* < 0.05).

### 2.2. Immune Enzyme Activity and Relative Expression of Immune-Related Genes

**Figure 1:**
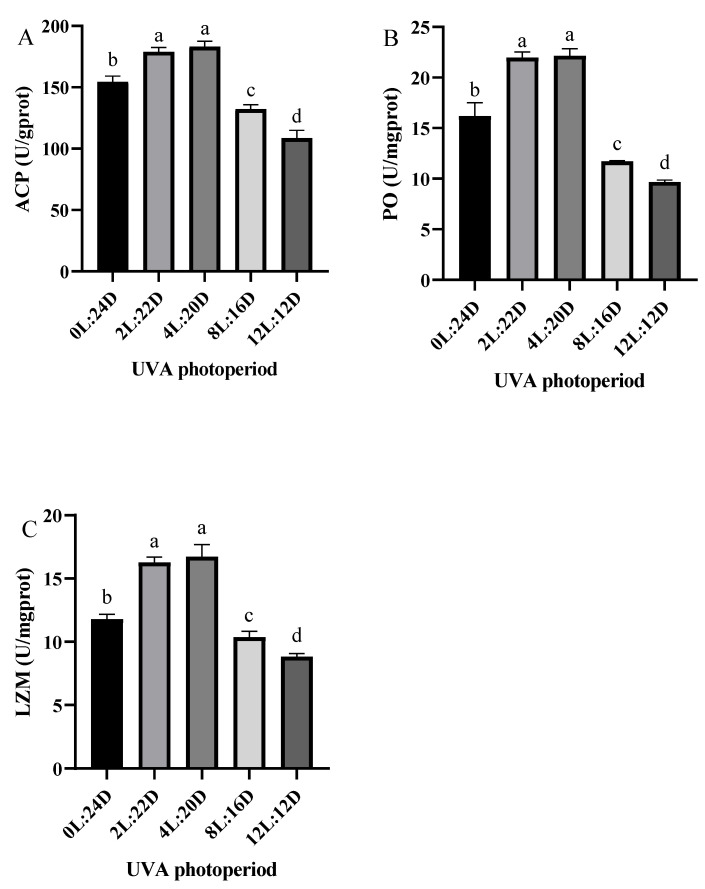
Hepatopancreas immune responses of *P. vannamei* to different UVA photoperiods for 28 days, including the immune enzyme activity of: ACP (**A**); PO (**B**) and LZM (**C**). Data are shown as mean ± SD (*n* = 3). Different lowercase letters illustrate significant differences among groups (*p <* 0.05).

The trend of the ACP, PO and LZM activities in hepatopancreas were consistent (Figure 1). They were the highest in 2L:22D and 4L:20D groups (*p* < 0.05). However, the 2L:22D and 4L:20D groups showed no significant differences. The activities of ACP, PO and LZM in the 8L:16D and 12L:12D groups were significantly lower than in the 0L:24D group (*p* < 0.05).

**Figure 2:**
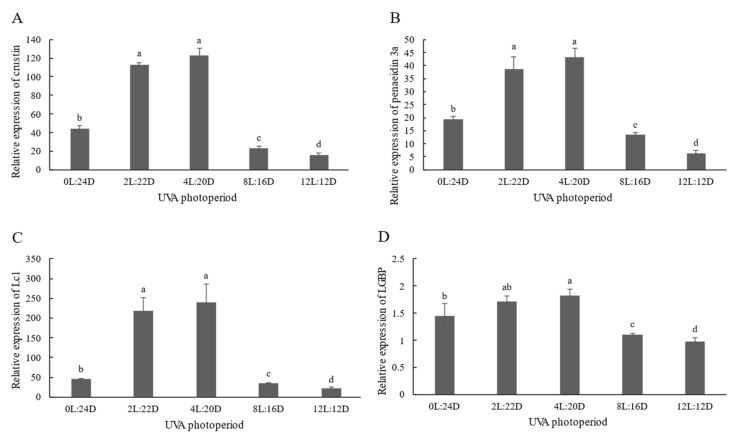
Hepatopancreas immune responses of *P. vannamei* to different UVA photoperiods for 28 days, including the mRNA expressions of: crustin (**A**); penaeidin 3a (**B**); Lc1 (**C**); and LGBP (**D**). Values are expressed as mean ± SD, from triplicate groups. Different lowercase letters illustrate significant differences among groups (*p* < 0.05).

The mRNA expressions of crustin, penaeidin 3a, Lc1 and LGBP in the 4L:20D group were significantly higher than in the other groups (*p* < 0.05) (Figure 2), except for the 2L:22D group. The relative expression levels of these genes in 8L:16D and 12L:12D were significantly lower than the control (*p <* 0.05).

### 2.3. Antioxidant Capacity and Relative Expression of Apoptosis-Related Genes

**Figure 3:**
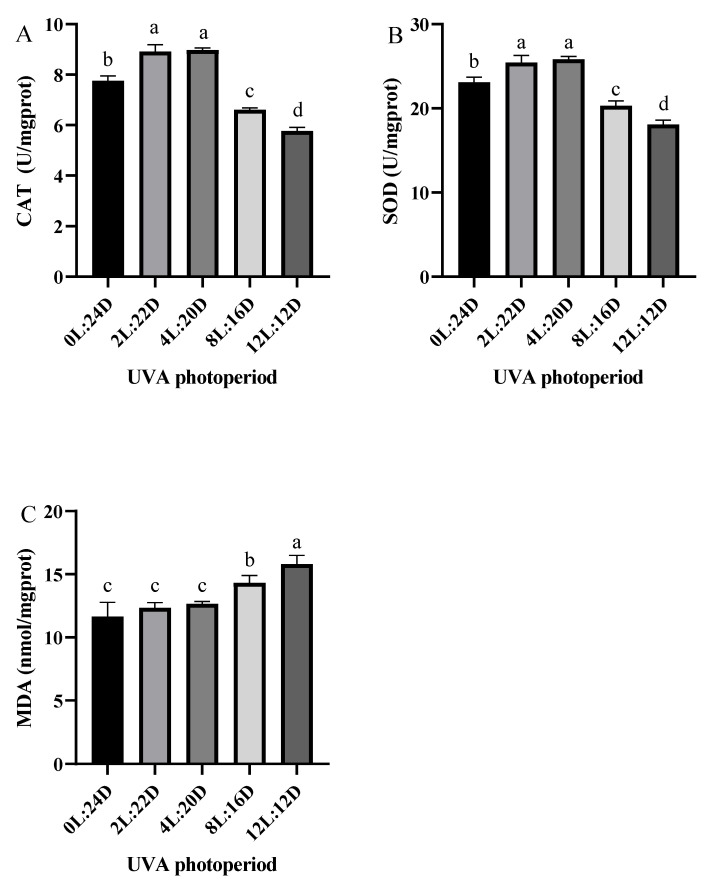
Hepatopancreatic antioxidant capacity of *P. vannamei* to different UVA photoperiods for 28 days, including of: CAT (**A**); SOD (**B**) and MDA (**C**). Values are expressed as mean ± SD, from triplicate groups. Different lowercase letters illustrate significant differences (*p* < 0.05) among groups.

The antioxidant responses of juvenile shrimp exposed to UVA are shown in Figure 3. The activities of SOD and CAT in hepatopancreas in the 2L:22D and 4L:20D were significantly higher than the other groups (*p* < 0.05). Furthermore, the activities of SOD and CAT in 8L:16D and 12L: 12D were also significantly reduced compared with the control group (*p* < 0.05). In addition, the MDA contents were increased with the extension of UVA irradiation time.

**Figure 4:**
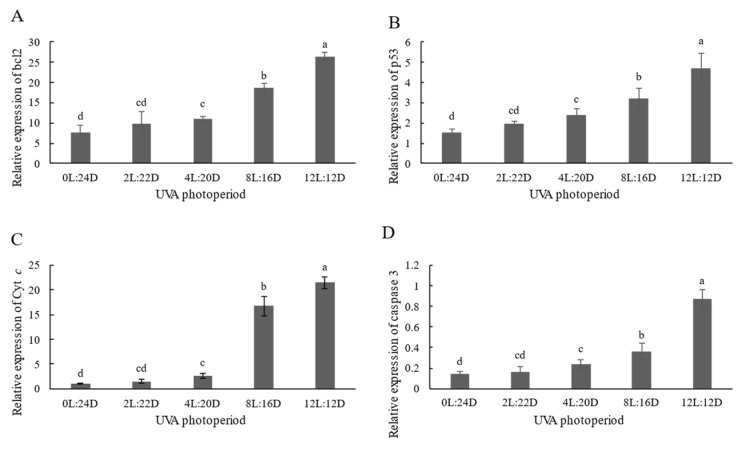
Hepatopancreas immune responses of *P. vannamei* to different UVA photoperiods for 28 days, including the mRNA expressions of: bcl2 (**A**); p53 (**B**); Cyt *c* (**C**); and Caspase 3 (**D**). Values are expressed as mean ± SD, from triplicate groups. Different letters illustrate significant differences (*p* < 0.05) among groups.

The mRNA levels of apoptosis-related genes (bcl2, p53, Cyt *c* and caspase 3) were significantly upregulated in the juvenile shrimp hepatopancreas exposed to 4L:20D, 8L:16D and 12L: 12D exposure treatment compared to the control group (*p* < 0.05) (Figure 4), whereas there was no significant difference between 2L:22D and the control group.

## 3. Discussion

### 3.1. Growth Performance

UV light is a vital component of the natural spectrum and serves a variety of ecological roles. UVA is the most common type of solar UV radiation in aquatic environments, whereas UVB is a source of environmental stress for many aquatic consumers [14]. However, the adjacent UVA band, which features many overlaps and interactions with UVB, has been neglected. UV radiation has been demonstrated to affect the growth and feeding of marine ectotherms such as fish and invertebrates in previous research. Key et al. [15] found that early life stage exposure to thin oil sheens and UV rays may have long-term deleterious effects on individuals and, eventually, grass shrimp populations. Short- and long-term UVB radiation caused negative consequences in juvenile European seabass (*Dicentrarchus labrax*), according to Ricardo et al. [16], and some of these impacts were accumulated and dose-dependent. Jordan et al. [17] found that cichlid feeding rates were increased by UVA. Thus, adding UV could exert a variety of effects on aquatic species, with each effect being species-specific. In this study, the WGR and SGR in the 2L:22D and 4L:20D groups were significantly higher, while the FCR in the 2L:22D and 4L:20D groups were significantly lower, compared with the other groups. This might be construed to mean that adding a particular quantity of UV to a shrimp’s environment increases feeding and, as a result, shrimp growth. Furthermore, we discovered that the shrimp in the 4L:20D group grew faster than those in the 2L:22D group, suggesting that *P. vannamei* prefers this UVA photoperiod. UV-induced genetic changes have been found in studies to have a deleterious impact on ontogenetic development [18], as when compared to individuals exposed to natural sun conditions, Vitt et al. [19] discovered that exposing stickleback *Gasterosteus aculeatus* to UVB radiation for an extra 4 h each day resulted in decreased growth and loss of body condition. We found similar trends in prawns. In this study, the WGR and SGR in the 8L:16D and 12L:12D groups were significantly lower than in the 0L:24D group; furthermore, the FCR and the mortality were both significantly higher. Our findings backed up a prior study, which found that excessive UV exposure inhibited growth to some extent, resulting in a high mortality rate.

### 3.2. Effects of Five UVA Photoperiod on Immune Responses

The innate immune system is critical in protecting the body against infections [20]. In contrast to vertebrates, shrimp possess a prophenoloxidase-activating system (proPO-AS), lectin agglutination and immune factor production, including antimicrobial peptide, lysozyme and hemolysin, while lectin and LGBP also play a major role in shrimp innate immunity. ACP is involved in phosphate hydrolysis and the lysosomal digestion of invading organisms during the metabolic process [21]. Additionally, the activities of ACP, PO and LZM in the 2L:22D and 4L:20D groups were significantly higher than the control. This suggests that appropriate UVA photoperiods can enhance the immune response of shrimp and improve their resistance to pathogens. Middlemiss et al. [22] reported that UV provided more beneficial properties to European lobster larvae than probiotic *Bacillus* spp. However, the long UVA photoperiod groups (8L:16D and 12L:12D) produced the opposite result, that the activities of these enzymes were lower than in other groups. This could be related to immunosuppression or immunological weariness brought on by prolonged exposure to stimuli [23]. Additionally, the shrimp in the 2L:22D and 4L:20D groups showed significantly higher crustin, penaeidin 3a and Lc1 expression values than the control. The value of LGBP expression was highest in the 4L:20D group. Vitt et al. [19] found that in levels of ambient UVB that were excessively increased resulted in a considerable negative effect on immunity in three-spined sticklebacks. When compared to the UVB-normal group, the UVB-enhanced fish demonstrated inferior development and body condition, as well as a lower splenosomatic index. Furthermore, fish exposed to UVB demonstrated a higher granulocyte-to-lymphocyte ratio, indicating that innate immunity was activated more than adaptive immunity. Furthermore, the expression levels of four genes were significantly lower in the 8L:16D and 12L:12D groups than in the other groups. Our results illustrate that short-duration UVA irradiation could enhance immune response and that prolonged UVA irradiation immune function was suppressed.

### 3.3. Effects of Five UVA Photoperiods on Antioxidant Enzyme Activity

Oxidative stress appears to be a prevalent environmental stress mechanism. The equilibrium between the formation of reactive oxygen species (ROS) and antioxidant defense is always disrupted when stressed [24,25,26]. UV radiation has already been proven to cause oxidative stress in cells and tissues by triggering a cascade of redox processes that produce ROS, and it also could cause DNA damage [6,27]. Antioxidant enzymes are essential for the elimination of deleterious ROS [25]. In this study, the activities of CAT and SOD in the short UVA photoperiod groups (2L:22D and 4L:20D) were significantly higher than those in the other groups. The MDA levels in the 12L:12D were the highest among all the groups. Increased levels of these enzymes in this study would aid in the elimination of ROS produced by the lighting environment. According to previous research, most crustaceans feature a high content of carotenoid [28], which performs essential functions in the immune and antioxidant systems [29,30] and may regulate the deleterious pro-oxidative consequences of increased UV exposure [31]. Our findings suggest that under the protection of carotenoids, particularly 2L:22D, a short UVA photoperiod may boost the antioxidant potential of shrimp to some extent. However, the long UVA photoperiod groups (8L:16D and 12L:12D) demonstrated the opposite result: the activities of these enzymes were lower than in other groups. We speculate that when it is exposed to prolonged UVA lighting, since the antioxidant system is unable to remove excessive ROS in a timely manner, oxidative damage and decreased enzyme function occur.

### 3.4. Effects of Five UVA Photoperiods on Apoptosis-Related Gene Expression

**Figure 5:**
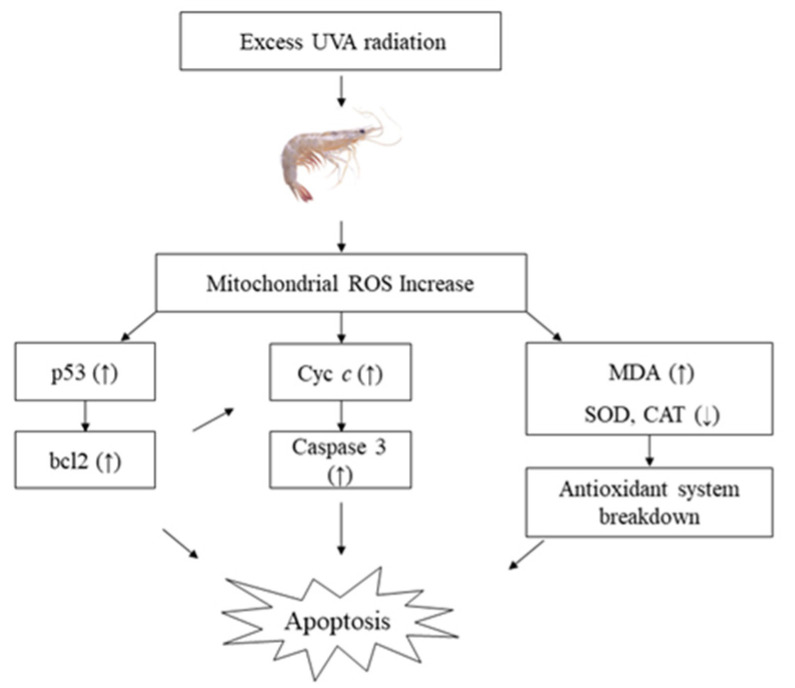
Prolonged UVA irradiation induces substantial stress, leading to apoptosis.

Oxidative stress has been extensively observed as a key inducer of apoptosis in a variety of cells [32,33]. Almost all metazoans use apoptosis to remove unwanted, contaminated or harmful cells [34]. It is a system for controlling the course of an immune response, as well as establishing immunological memory and central and peripheral tolerance. The caspase family is required for apoptosis to occur [35]. Caspase-3 is the major apoptosis executor among the caspase family members. The expression of caspase-3 in shrimp has been shown to be influenced by pathogen infection [36]. Cytochrome c (Cyt *c*) is a major signaling molecule for apoptosis (Hasini et al., 2019). Based on the control of apoptosis in vertebrates, both p53 production and transcription suggest cell cycle monitoring and a possible signal to induce apoptosis [37]. Studies performed by Hollmann et al. [38] have shown that in crustaceans, p53 causes apoptosis by activating anti- and pro-apoptotic molecules such as Bcl2. Many investigations on the effects of environmental pollutants on fish and shrimp have found indications of p53 activation inducing apoptosis [35,39]. Hangjun et al. [40] reported that mitochondrial Cyt *c* can be affected by changes in intracellular bcl2 expression patterns. Caspase 3 catalyzes the cleavage of several important cellular proteins [41], while the intrinsic pathway started by the Cyt *c* complex may further activate cascade effector caspase 3 [42]. In our study, we discovered that the expression levels of bcl2, p53, Cyt *c* and caspase 3 in the hepatopancreas of shrimp was not significantly different in 0L:24D and 2L:22D, whereas the other groups were significantly higher than the control. Furthermore, these increased significantly when the delay UVA photoperiod was delayed. According to certain studies, UV irradiation can cause the cleavage of a number of proteins, including nuclear lamins and fodrin, resulting in cell apoptosis [43,44]. Ideal shrimp health management for blunt snout bream would be founded on a rearing environment that provides the most favorable conditions for growth and survival. The results of the present study indicated that short-duration UVA irradiation does not significantly affect apoptosis, while prolonged UVA irradiation induces substantial stress, leading to apoptosis (Figure 5). Long UVA photoperiods (more than 8 h) not only produced poor growth, but may also have caused a stress response, which might consequently lead to elevated hepatopancreas oxidation rates and depress immunity in this species. Although the growth performance, different immune parameters, antioxidant status and apoptosis-related gene expression of shrimp in different UVA photoperiod have been studied, molecular mechanisms are scarce and need to be elucidated further in future studies. We plan to conduct transcriptomic studies to explore the mechanisms of action of UVA in *P. vannamei* in the future.

## 4. Materials and Methods

### 4.1. Experimental Shrimp

We performed this experiment at Yuhai Hongqi Ocean Engineering Co. Ltd., Rizhao, Shandong, China. Juvenile *P. vannamei* (initial wet weight: 9.56 ± 0.10 g) were purchased from a local *P. vannamei* farm in Rizhao, Shandong, China. All the shrimp utilized in the study appeared to be in good physical condition and showed no symptoms of trauma.

### 4.2. Experimental Design

The shrimp were placed in aquariums for 7 days before the experiment to adapt to the experimental settings. The shrimp were fed a commercial diet three times a day during the acclimatization period. The feeding was stopped 24 h before the experiment began. Each treatment included three tanks (length: 48 cm, breadth: 45 cm, height: 48 cm, effective water volume: 70 L), and each tank contained 30 shrimp. The experiment with shrimp was cultured in a recirculating aquaculture system (RAS). During the experiment, the shrimp were fed commercial feed three times per day to keep them completely nourished. The nutritional compositions of the basal diets are shown in Table 2. The feeding quantity was equal to 3% of the wet body weight of the shrimp. Remaining food and feces were removed after 1 h. The number of shrimp that died was counted in order to calculate the mortality rate. The water condition was maintained at 25.0 ± 2 °C, the salinity was 27–29‰, the pH was 7.5 ± 0.1 and concentration of dissolved oxygen was ≥8.0 mg/L. The levels of total ammonium nitrogen (TAN) and nitrite in all the treatments were measured every two days using the techniques described by Ricart-Jané et al. [45] and Kim et al. [46] and kept at TAN ≤ 0.2 mg/L and nitrite ≤ 0.1 mg/L.

The lamp used in this study was designed by the Institute of Semiconductors, CAS and manufactured by Wuxi Huazhaohong Optoelectronic Technology Co., Ltd. (Wuxi, China) and Shenzhen Fluence Technology PLC (Shenzhen, China). A control group (full-spectrum treatment) and four full-spectrum treatments with different UVA (peak at 400 nm) photoperiod environments (2L: 22D, 4L: 20D, 8L: 16D and 12L: 12D) were used. The photoperiod of the full spectral light was 12L: 12D. A spectroradiometer (PLA-20 Plant Lighting Analyzer, Hangzhou, China) was used to measure the wave peak of the spectral compositions and light intensity 2 cm above the water surface after the light source was steady to ensure that the peaks stayed within the proper range. Each UVA treatment group received 1 W/m^2^ of light and the intensity of the full-spectrum light was 1 W/m^2^. The local UVA light intensity was measured every two hours from 6:00 to 18:00 and averaged to produce 50% of the experimental light intensity. To avoid light pollution, the various treatment groups were separated by black-out coverings.

### 4.3. Sample Collection

At the beginning and end of the experiment, the wet weights of all the shrimp were measured. After fasting for 24 h, six shrimp were randomly selected from each tank at the end of the experiment and anesthetized with tricaine mesylate (MS-222). The hepatopancreas of each shrimp was sampled on a pre-chilled culture dish maintained at 0 °C, then homogenized and centrifuged for 10 min at 4 °C at 2500 r, with the supernatant collected and preserved for enzyme activity testing. The other hepatopancreas were kept at 80 °C in RNA storage (Conchbio Co., Ltd., Qingdao, China) for subsequent RNA extraction and testing of immune and apoptosis-related genes.

### 4.4. Analysis of Enzyme Activity

Kits bought from Nanjing Jiancheng Bioengineering Institute (Nanjing, China) were used to measure catalase (CAT), superoxide dismutase (SOD), malonaldehyde (MDA), acid phosphatase (ACP), phenol oxidase (PO) and lysozyme (LZM) activities, according to the kit instructions.

### 4.5. Real-Time PCR Analysis

The Animal Total RNA Isolation Kit (Foregene, Chengdu, China) was used to extract total RNA from the hepatopancreas, followed by DNase I treatment, as instructed by the manufacturer. The quality and quantity were determined using a 1% agarose gel electrophoresis and spectrophotometric measurements at 260 nm and 280 nm. The whole RNA was then reverse-transcribed into cDNA, according to the manufacturer’s instructions, using a PrimeScriptTM RT Master Mix (Perfect Real Time) (Takara, Dalian, China). Until further analysis, the cDNA templates were kept at 80 °C.

Table 3 lists the genes and primers used in the gene expression analysis. TB Green^®^ *Premix Ex Taq*^TM^ II (Tli RNaseH Plus) was used in real-time PCR analysis on the ABI PRISM 7500 Detection System (Applied Biosystems, Foster City, CA, USA) (Takara, Dalian, China). According to normal methods, real-time PCR was performed for bcl2, p53, Cyt *c*, caspase-3, crustin, penaeidun 3, Lc1, LGBP and housekeeping gene (β-actin) using the primer sequences listed in Table 1. In total, 10 μ TB Green *Premix Ex Taq* II Tli RNaseH Plus (2×), 6 μL sterile water, 2 μL cDNA, 0.8 μL PCR forward primer (10 umol/mL), 0.8 μL PCR reverse primer (10 umol/mL) and 0.6 μL ROX reference dye (50×) were used in the reaction system. A two-step quantitative PCR reaction method was used, with predenaturation at 95 °C for 30 s, denaturation at 95 °C for 5 s and annealing at 60 °C for 30 s (40 cycles). At 65–95 °C, the solubility curve was constructed with a measurement every 0.05 °C. All of the samples were analyzed in parallel with the housekeeping gene to standardize cDNA loading. The relative gene expression was calculated using 2^−ΔΔCt^ after verification that the primers were amplified with an efficiency of approximately 100%, as expatiated by Livak and Schmittgen [47].

### 4.6. Data Processing and Analysis

Growth rate (%) = 100% × (W_t_ − W_0_)/W_0_

Mortality (%) = 100% × [(N_0_ − N_t_)/N_0_]

SGR (%) = 100% × (LnW_t_ − LnW_0_)/t

FCR = F/(W_t_ − W_0_)

Where N_t_ and N_0_ are the final and initial body number, W_t_ and W_0_ are the final and initial body weight (g), t is time of rearing (days) and F is total feed intake (g).

Using SPSS 26.0 software, the results were subjected to one-way analysis of variance (ANOVA), followed by a Duncan’s test to determine significance among the shrimp groups (SPSS Inc., Chicago, IL, USA). Unless otherwise noted, significant values were established at *p* < 0.05 for all the analyses. Means and standard deviations are used to represent all the data.

## 5. Conclusions

The growth performance, immune responses, antioxidant status and apoptosis-related gene expression of *P. vannamei* under five different UVA photoperiods in full-spectrum background light (12L:12D) were investigated. Exposure to 2L:22D and 4L:20D UVA photoperiods resulted in increased growth performance and immune responses while decreasing stress responses, whereas the opposite results were found for the long UVA photoperiod groups (8L:16D and 12L:12D). The apoptosis-related gene expression levels in the 2L:22D and 4L:20D groups were lower than with the other treatments, except for the control group (0L:24D). As a consequence, supplementing with UVA light at specific periods activated antioxidant enzyme activity and the immunological defense system, protecting cells from oxidative stress. We believe that selecting a specific UVA photoperiod is beneficial to shrimp growth and immunological function and that the effects of choosing 2L: 22D and 4L:20D UVA photoperiods are considerable, based on the results of this experiment.

## Figures and Tables

**Table 1:** Growth rate, feed conversion ratio (FCR), specific growth rate (SGR) and mortality of *P. vannamei* in the five light environments.

UVA Photoperiod	Initial Weight (g)	Final Weight (g)	Growth Rate (%)	FCR (%)	SGR (%)	Mortality (%)
0L:24D	9.60 ± 0.04	17.68 ± 0.46 ^b^	84.13 ± 5.59 ^b^	1.17 ± 0.14 ^c^	2.18 ± 0.11 ^b^	26.67 ± 3.33 ^c^
2L:22D	9.55 ± 0.09	19.35 ± 0.64 ^a^	102.69 ± 6.28 ^a^	0.86 ± 0.04 ^d^	2.52 ± 0.11 ^a^	18.89 ± 1.92 ^d^
4L:20D	9.62 ± 0.17	19.68 ± 0.40 ^a^	104.78 ± 6.89 ^a^	0.74 ± 0.02 ^d^	2.56 ± 0.12 ^a^	17.78 ± 1.92 ^d^
8L:16D	9.53 ± 0.05	16.14 ± 0.17 ^c^	69.44 ± 1.88 ^c^	1.59 ± 0.08 ^b^	1.88 ± 0.04 ^c^	35.56 ± 3.85 ^b^
12L:12D	9.51 ± 0.12	14.73 ± 0.67 ^d^	54.79 ± 5.16 ^d^	2.34 ± 0.22 ^a^	1.56 ± 0.12 ^d^	43.33 ± 5.77 ^a^

Note: The data are expressed as the mean ± SD. Different lowercase letters indicate significant differences among groups (*p* < 0.05).

**Table 2:** Nutritional compositions of basal diets.

Ingredients	Content (%)
Crude protein	43.25%
Crude fat	7.41%
Crude fiber	3.76%
Crude ash	13.24%
Moisture	11.88%
Total phosphorus	1.05%
Lysine	2.43%

**Table 3:** Primers used for qRT-PCR analysis.

Gene	Accession No.	Primer
bcl2	MH559339.1	F: ATGTTGCTGTGCACCAAGTG
		R: AAGGCAGCACATGAACACGA
p53	KX179650.1	F: GTGGAAGTGTTGCCAAGCAG
		R: CGAATTTGTGACGACCTGCC
cytochrome C (Cyt *c*)	KX096890.1	F: CGTACACGTCCAGCAAAAGC
		R: GGTGTACACGTAGCCTGGTG
caspase-3	EU421939.1	F: GGTGGACAAAGGCGTGAGTA
		R: CTCGGCCAAGAAGTGGATGA
crustin	AY486426.1	F: ACCTGTTCCAACGGCTACAA
		R: AACCTGCGATCCGAGGAATG
penaeidin 3a	AF390139.1	F: GCCGGGGAATTTCCTTCTCA
		R: ACAGGTTGTCAAGCGAGGTT
C-type lectin (Lc1)	KY937940.1	F: AGCTGGCACGAAAGACATCA
		R: GAGACACCGCTCGTCGTTAT
LGBP	EU102286.1	F: CGTCTCCGAACCATGTCCAA
		R: CAAAGTTGTCGTTGCCCCTG
β-Actin	AF300705.2	F: CTCGCAGTCCAACCCGAG
		R: TCTACAACCAGGGCGGCTA

## Data Availability

All data are reported in the manuscript.
